# Relationship between hyperglycemia, hormone disturbances, and clinical evolution in severely hyperglycemic post surgery critically ill children: an observational study

**DOI:** 10.1186/1472-6823-14-25

**Published:** 2014-03-14

**Authors:** Yolanda Ballestero, Jesús López-Herce, Rafael González, Maria José Solana, Jimena del Castillo, Javier Urbano, Marta Botran, Ana García, Nieves López, Jose María Bellón

**Affiliations:** 1 Pediatric Intensive Care Department, Hospital General Universitario Gregorio Marañón Complutense University of Madrid, Madrid, Spain; 2 Laboratory Department, Hospital General Universitario Gregorio Marañón, Madrid, Spain; 3 Instituto de Investigación biomédica del Hospital Gregorio Marañón, Madrid, Spain; 4 Red de Salud Materno Infantil y del Desarrollo, Red SAMID II, Madrid, Spain

**Keywords:** Hyperglycemia, Critically ill children, Length of stay, Hormone disturbances

## Abstract

**Background:**

To study hormonal changes associated with severe hyperglycemia in critically ill children and the relationship with prognosis and length of stay in intensive care.

**Methods:**

Observational study in twenty-nine critically ill children with severe hyperglycemia defined as 2 blood glucose measurements greater than 180 mg/dL. Severity of illness was assessed using pediatric index of mortality (PIM2), pediatric risk of mortality (PRISM) score, and pediatric logistic organ dysfunction (PELOD) scales. Blood glucose, glycosuria, insulin, C-peptide, cortisol, corticotropin, insulinlike growth factor-1, growth hormone, thyrotropin, thyroxine, and treatment with insulin were recorded. β-cell function and insulin sensitivity and resistance were determined on the basis of the homeostatic model assessment (HOMA), using blood glucose and C-peptide levels.

**Results:**

The initial blood glucose level was 249 mg/dL and fell gradually to 125 mg/dL at 72 hours. Initial β-cell function (49.2%) and insulin sensitivity (13.2%) were low. At the time of diagnosis of hyperglycemia, 50% of the patients presented insulin resistance and β-cell dysfunction, 46% presented isolated insulin resistance, and 4% isolated β-cell dysfunction. β-cell function improved rapidly but insulin resistance persisted. Initial glycemia did not correlate with any other factor, and there was no relationship between glycemia and mortality. Patients who died had higher cortisol and growth hormone levels at diagnosis. Length of stay was correlated by univariate analysis, but not by multivariate analysis, with C-peptide and glycemic control at 24 hours, insulin resistance, and severity of illness scores.

**Conclusions:**

Critically ill children with severe hyperglycemia initially present decreased β-cell function and insulin sensitivity. Nonsurvivors had higher cortisol and growth hormone levels and developed hyperglycemia later than survivors.

## Background

The incidence of hyperglycemia is very high, between 56 and 86%, and it is an independent risk factor for morbidity and mortality in critically ill patients [1-3].

Many studies have analyzed the effect of treatment of hyperglycemia on the prognosis of critically ill patients. Although initial studies found that intensive insulin treatment improved the prognosis, subsequent research has not confirmed these findings and has detected a higher incidence of hypoglycemia [1-7].

Few studies have investigated the pathophysiological mechanisms involved in hyperglycemia of the critically ill patient [8-11]. Hyperglycemia of critically ill adults and children have different characteristics. An understanding of the mechanisms that give rise to hyperglycemia in the critically ill child could help to define the most appropriate treatment.

The objectives of the present study were to investigate the pathophysiological mechanisms of severe hyperglycemia in the critically ill child, to analyze the clinical factors associated with hyperglycemia, and to evaluate the association of hyperglycemia with prognosis and length of hospital stay.

## Methods

A prospective observational study was performed in patients aged between 1 month and 16 years admitted to the Pediatric Intensive Care Unit (PICU) between July 2009 and March 2010 and who developed significant hyperglycemia, defined as 2 blood glucose measurements greater than 180 mg/dL, separated by at least 2 hours, at any time during admission to the PICU. Children with type 1 diabetes mellitus were excluded from the study.

The study was approved by the Investigation and Ethics Committee of the Gregorio Marañón Hospital. Parents or legal guardians gave their parental permission for the child to participate in the study and to publish the results.

Clinical severity was assessed using the pediatric index of mortality 2 (PIM2) [12], pediatric risk of mortality (PRISM) score [13], and pediatric logistic organ dysfunction (PELOD) scale [14]. PIM score calculates the risk of mortality based in the first clinical and analytical data after admission. They include the kind of admission (programmed or no), the diagnosis classified in high and low-risk, photomotor reflex, mechanical ventilation, blood pressure, base deficit and FiO2/PaO2 [12]. PRISM score calculates the risk of mortality based on the worst values in the first 24 h of admission of several physiological values (heart rate, blood pressure, Glasgow scale and pupillary reaction) and analytical values (PaO2/FiO2, PaCO2, prothrombine time, billirubin, calcium, potassium, glycemia) [13]. PELOD score evaluates the risk of mortality analyzing the number of failure of different systems by different parameters: cardiovascular (heart rate, blood pressure), respiratory (PaO2/FiO2, PaCO2, mechanical ventilation), neurologic (Glasgow score, pupillary reactions), hematologic (leucocytes, platelets), renal (creatinine), hepatic (AST, prothrombine time). Scores was recorded at the time of diagnosis of hyperglycemia and 24 and 72 hours later. Treatment with corticosteroids, the doses of vasoactive drugs, the vasoactive score calculated by the following formula: dopamine dose (μg/kg/min) + dobutamine dose (μg/kg/min) + 100 epinephrine dose (μg/kg/min) + 10 milrinone dose (μg/kg/min) + 10.000 vasopresine dose (U/kg/min) + 100 noerpinephrine dose (μg/kg/min) [15], blood glucose, glycosuria, insulin, C-peptide, cortisol, corticotropin (ACTH), insulinlike growth factor (IGF-1), growth hormone (GH), thyrotropin (TSH), free thyroxine (T_4_), and dose of insulin treatment were recorded at the time of diagnosis of hyperglycemia and 24 and 72 hours later.

Insulin, C-peptide, cortisol, and IGF-1 were measured using a solid-phase chemoluminescent enzyme immunometric assay (Immulite 2000, Siemens), ACTH and GH using an immunoradiometric assay (DiaSorin), TSH using a solid-phase paramagnetic-particle chemoluminescent enzyme immunometric assay (Access®Beckman Coulter™), and free T_4_ using a 2-step, paramagnetic-particle competitive chemoluminescent enzyme immunoassay (Access®Beckman Coulter™).

β-cell function and insulin sensitivity and resistance were determined on the basis of the homeostatic model assessment (HOMA) calculated by computer software (http://www.OCDEM.ox.ac.uk) [16,17]. HOMA model is used to estimate insulin sensitivity (HOMA-%S) and β-cell function (HOMA-%B) using blood glucose and C-peptide levels [16]. Normal β-cell function is a HOMA-%B of 100%, normal insulin sensitivity is a HOMA-%S of 100%, and normal insulin resistance is 1 [16].

In accordance with our protocol, insulin was administered to those patients who presented blood glycemia above 200 mg/dL in 2 measurements separated by 4 hours. Treatment was started with 0.05-0.1 IU/kg/h, with hourly monitoring of the glycemia and glycosuria and adjustment of the insulin infusion to achieve glycemia between 120 and 150 mg/dL; subsequent monitoring was performed at least every 4 hours and the insulin infusion was interrupted if the glycemia fell below 120 mg/dl. The dose and duration of insulin treatment and the adverse effects attributable to treatment were recorded. Hypoglycemia was defined as glycemia less than 60 mg/dL.

The presence of infection, the need for and duration of mechanical ventilation, continuous renal replacement therapy (CRRT), and extracorporeal membrane oxygenation (ECMO), the duration of PICU admission, and mortality and its causes were also recorded.

Statistical analysis of the results was performed with the SPSS statistical package, version 18. The nonparametric Wilcoxon, Mann–Whitney and Kruskal-Wallis tests were used to compare quantitative variables, the Pearson *χ*^2^ test was used to compare frequencies, and the nonparametric Spearman rank correlation (ρ) was used to determine the correlation between quantitative variables. A *P* value less than .05 was considered significant. Multiple logistic regression analysis was made to study the association of variables with length of PICU admission and mortality.

## Results

### Characteristics of patients

Thirty-one hyperglycemic episodes were detected in 29 patients (2 patients presented 2 episodes separated by more than 7 days), aged between 1 and 204 months (median, 36 months) and weighing between 4 and 70 kg (median, 16.3 kg).

The reasons for admission were the following: postoperative period of cardiac surgery in 22, postoperative period of colectomy for ulcerative colitis in 1, cardiogenic shock in 4 and respiratory disease in 2.

Mechanical ventilation was required by 74% of patients, ECMO by 41%, and renal replacement therapy by 41% during admission to the PICU, and 48% developed nosocomial infection (respiratory tract in 24%, sepsis in 17%, and other sites in 6%). 96.5% of patients received vasoactive drugs (initial vasoactive score was 3.5 (IQR 3–5) and it did not change during the study. Four patients (13%) died during admission to the PICU, 3 due to multiorgan failure and 1 due to cerebral hemorrhage.

Initially all patients received intravenous glucose infusion and during the study enteral nutrition was started and increased progressively. Carbohydrate delivery is showed in Table 1. None of patients received parenteral nutrition.

**Table 1:** Changes in hormone levels and clinical parameters

	**At diagnosis of hyperglycemia**		**At 24 hours**		**At 72 hours**		***P***
	**Median**	**(IQR)**	**Median**	**(IQR)**	**Median**	**(IQR)**	
**Blood glucose**, mg/dl	249	217-315	153	112-194	125	109-166	0.0001
**Insulinemia**, mUI/L	20	8.5-32.5	13	6.5-27.5	16	7-29	0.401
**C-peptide**, nmol/L	2	0.8-2.7	1.6	1-2.6	2	0.9-3.9	0.903
**β-cell function with C-peptide** %	49.2	30.8-91.3	96.3	78.4-139.8	135.9	95.2-187.8	0.001
**Insulin sensitivity % with C-peptide**	13.2	10.3-32.2	21.9	11.9-33.0	20.6	11.9-38.7	0.085
**Insulin resistance with C-peptide**	7.6	3.1 -9.6	4.5	3-8.4	4.9	2.7-8.3	0.081
**Carbohydrate delivery**, g/kg/day	2	1.8 -4.4	4.8	1.9-8.5	7.3	4.8-9.2	0.001
**TSH,** mUI/L	0.5	0.3-0.8	0.3	0.2-0.9	0.6	0.2-3.3	0.091
**Free T4**, ng/dL	1	0.8-1.2	0.9	0.7-1.2	0.7	0.5-1.2	0.009
**ACTH,** ng/L	21.5	12.8-41	21	12-33.5	20.8	11.6-34.4	0.410
**Cortisol**, μg/dL	43.7	12.6-77.3	16.1	7.9 -30.1	15.5	8.9-34.9	0.001
**GH,** μg/L	0.8	0.5-7.1	1.2	0.5-2.9	1.8	0.6-3.4	0.708
**IGF1,** μg/L	26	25-89.	25	25-54	25	25-48	0.132
**PRIMS** %	15.6	5.7-42.5	13.2	4.2-33.6	7.5	1.4-29.2	0.349
**PIM2** %	2.7	1.4-11.8	3.4	2-18.9	5.4	0.5-25.2	0.397
**PELOD** %	6.7	1-19.5	1.5	0.1-1.7	1.3	0-2.3	0.787
**HR**, bpm	125	113.7-150.2	130	112.5-150	127.5	110-148.5	0.482
**MAP**, mmHg	62	55.5-72.2	67	54.5-73.5	63	58-79.5	0.837

*Abbreviations*: *ACTH* corticotrophin, *bpm* beats per minute, *GH* growth hormone, *HR* heart rate, *IGF* insulinlike growth factor, *IQR* interquartile range, *MAP* mean arterial pressure, *PELOD* pediatric logistic organ dysfunction, *PIM2* pediatric index of mortality 2, *PRIMS* pediatric risk of mortality, *T4* thyroxine, *TSH* thyrotropin.

### Evolution of blood glucose and hormone levels

The changes in the blood glucose, hormone levels, clinical severity scores, and clinical parameters over the 72 hours of the study are shown in Table 1. The median initial blood glucose was 249 mg/dL, and this fell significantly to reach values of 125 mg/dL at 72 hours, despite a progressive increase in carbohydrate ingestion (Table 1). Intravenous insulin treatment was required in 83% of the episodes (mean dose, 0.11 ± 0.09 U/kg/h; mean duration of treatment, 45.2 ± 74.6 hours). The minimum blood glucose was 74 mg/dL. None of the patients developed hypoglycemia.

The insulin and C-peptide levels were elevated initially in 64% and 72% of patients, respectively, and did not show significant variations over the 72 hours of the study. β-cell function and insulin sensitivity were initially low but increased over the first 24 hours, although only the change in β-cell function reached statistical significance.

At the time of diagnosis of hyperglycemia, 50% of the patients presented insulin resistance and β-cell dysfunction, 46% presented isolated insulin resistance, and 4% isolated β-cell dysfunction.

Cortisol levels were initially elevated (levels above 25 μg/dL in 64% of patients), though ACTH levels were normal. During the course of the study, there was a significant fall in cortisol levels but no change in ACTH. Patients did not received steroids during the three days of the study. 15 patients received steroids previously to the diagnosed of hyperglycemia. There were no significant differences in initial cortisol levels of patients treated with steroids (47.3 μg/dL) than of the rest of patients (57.9 μg/dL).

T_4_ levels were initially normal, whereas 44% of patients presented low TSH levels. Over the course of the study there was a significant fall in T_4_ (36% of patients had low T_4_ levels at 72 hours), though there were no significant changes in TSH levels.

The levels of GH and IGF-1 were initially low and there were no significant changes over the course of the study.

### Correlation between parameters

No relationship was observed between the blood glucose at any time during the evolution and most of hormone parameters, clinical variables, and treatments. Nor were there differences in the changes in blood glucose over the course of the study according to age, sex, diagnosis, need for mechanical ventilation, ECMO or CRRT, or the presence of nosocomial infection.

Only at 24 and 72 hours there was a significant correlation between the blood glucose and insulin and C-peptide levels and between these 3 parameters and the PRIMS, PIM2, and PELOD clinical severity scores (data not showed). There was a relationship between the maximum blood glucose and the maximum dose of insulin administered (ρ = 0.596, *P =* .001), but not with the duration of insulin treatment.

### Correlation with prognosis

There was no relationship between glucose levels over the course of the study and mortality. However, the onset of hyperglycemia occurred later in nonsurvivors than in survivors. The following factors were associated with mortality: cardiogenic shock (*P* = .014), nosocomial infection (*P* = .008), and the need for ECMO (*P* < .001) or CRRT (*P* < .001). Compared to survivors, patients who died had higher cortisol levels at the time of diagnosis of hyperglycemia and 24 hours later. GH values at the time of diagnosis of hyperglycemia were also higher in the patients who died. Compared to survivors, patients who died presented greater insulin resistance 72 hours after diagnosis (Table 2). There was no relationship between mortality and the values of the other hormones. Multiple logistic regression analysis could not be performed because only four patients died.

**Table 2:** Comparison between survivors and nonsurvivors

	**Survivors**		**Nonsurvivors**		***P***
	**Median**	**(IQR)**	**Median**	**(IQR)**	
Onset of hyperglycemia, h	6.5	(3.7 -27)	59	(13.5-137.5)	.028
Baseline cortisol, μg/dL	37	(12.4-66)	100	(74–138)	.024
Cortisol at 24 h, μg/dL	13	(5.6 -22.4)	39.7	(29.9-59.3)	.003
Baseline GH, μg/dL	0.7	(0.5-3.6)	15	(1.8-31)	.026
Sensitivity with C-peptide at 72 h, %	29.0	(14.3-67.8)	11.9	(10.7-12.9)	.026
IR with C-peptide at 72 h	3.4	(1.5-6.9)	8.4	(7.7 -9.3)	.026
Cardiogenic shock	7.3%		60%		.014
Nosocomial infection	30.7%		100%		.008
ECMO	0%		100%		.001
CRRT	0%		100%		.001

*Abbreviations*: *CCRT* continuous renal replacement therapy, *ECMO* extracorporeal membrane oxygenation, *GH* growth hormone, *IR* insulin resistance, *IQR* interquartile range.

The mean length of stay in the PICU was 16.4 ± 14.8 days. Length of stay was associated with the severity of illness scores, the blood glucose, C-peptide levels, insulinemia (Table 3), the time from admission to the onset of hyperglycemia (r = 0.532, p = 0.002), the maximum dose of insulin (r = 0.539, p = 0.004), and the duration of treatment with insulin (r = 0.602, p = 0.001). There were also statistically significant correlations between the length of PICU stay and the presence of nosocomial infection (*P* = .001) or the need for ECMO (*P* = .012), CRRT (*P* = .012), or mechanical ventilation (*P* = .001).

**Table 3:** Correlation between length of pediatric intensive care stay and other variables

	**Time of diagnosis of hyperglycemia**		**24 h after the diagnosis**		**72 h after diagnosis**	
	**Correlation (r)**	***P***	**Correlation (r)**	***P***	**Correlation (r)**	***P***
**PRIMS**			0.508	0.060	0.480	0.037
**PIM2**	0.568	0.004	0.626	0.001	0.548	0.015
**PELOD**	0.499	0.013	0.497	0.007	0.615	0.005
**MAP**	−0.429	0.018			−0.515	0.020
**Blood glucose**			0.531	0.004		
**C-peptide**			0.697	0.001	0.618	0.002
**Insulinemia**			0.542	0.030		
**IGF-1**	−0.500	0.011	−0.393	0.043		

Multiple logistic regression analysis did not identify factors associated with a length of PICU stay more than 14 days, possibly due to the low number of patients studied. The univariate and multivariate analyses of the patients in the postoperative period of cardiac surgery did not show different results than analysis of the total population (data not shown).

## Discussion

Critical illness hyperglycemia (CIH) is probably due to endogenous counter-regulatory hormones and other diabetogenic factors, such as proinflammatory mediators and therapeutic interventions, including the administration of glucocorticoids and catecholamines, which interfere with insulin-receptor signaling and/or glucose transport and utilization within cells [8-11,18-21]. On the other hand insulin therapy acts to suppress counterregulatory hormones and proinflammatory transcription factors.

Our study is the first to analyze the relationship of severe hyperglycemia in the critically ill child with the values of various hormones and with the clinical course.

### Mechanisms of production of hyperglycemia and relationship with hormonal alterations

Hyperglycemia is a state of metabolic dysregulation due to an imbalance between insulin production and insulin sensitivity in the target tissues [8].

#### C-peptide and insulin levels

Studies in adults suggest that CIH is primarily due to insulin resistance in spite of a situation of supranormal β-cell function, because beta cell could not secrete as much insulin as that necessary to compensate insulin resistance [17,22]. However, in CIH in children, the C-peptide levels may be low or elevated [8]. Our patients presented elevated C-peptide levels, although these levels were relatively low given the blood glucose levels at that time, which would suggest the presence both of reduced β-cell function and of increased insulin resistance.

Preissig et al., found in 41 critically ill children with hyperglycemia, that low levels of insulin were associated with a rapid onset of hyperglycemia, greater clinical severity, greater insulin requirements and increased duration of mechanical ventilation and length of PICU stay [8]. We found higher C-peptide levels in more severely ill patients (higher PRISM, PIM2, and PELOD scores) and that higher C-peptide levels were associated with a longer PICU stay, probably because C-peptide levels were a marker of more severe ill. In contrast to the findings of Preissig et al., in our study the onset of hyperglycemia occurred later in the more seriously ill patients.

In a study of children with meningococcal sepsis and hyperglycemia 62% presented isolated insulin resistance, 17% β-cell dysfunction and 21% combined β-cell dysfunction and insulin resistance [17]. In our patients, there was a reduction both in β-cell function and in insulin sensitivity at the time of diagnosis of the hyperglycemia. Both improved at 24 hours and β-cell function normalized at 72 hours, though a degree of insulin resistance persisted. This would suggest that the mechanism of hyperglycemia in critically ill children with severe hyperglycemia is probably multifactorial and involve both a relative reduction in insulin production due to beta cell dysfunction whose cause of the is not well known, and peripheral resistance to insulin.

It has been reported that treatment with exogenous insulin improves insulin sensitivity [23]. In our study there was a slight improvement in insulin sensitivity at 24 hours, coinciding with insulin treatment, though sensitivity continued to be low at 72 hours.

#### Inotropic index

Catecholamines may directly suppress β-cell function and insulin secretion [8]. Some authors have reported that the inotropic index is inversely related to C-peptide levels and that hyperglycemic children have a higher inotropic index [18]. In contrast, other authors have found no correlation between the inotropic index and blood glucose, insulin or C-peptide levels [17], as occurred in our study.

#### Corticosteroids

Corticotropin-releasing hormone and ACTH control cortisol secretion in the acute phase of critical illness, whereas non-ACTH-mediated pathways (interleukin [IL] 1, IL6, and tumor necrosis factor-α) are involved during prolonged critical illness. Protracted critical illness is associated with a fall in plasma ACTH concentrations despite the persistence of a state of hypercortisolism [24].

Our results, with very high initial cortisol levels, coincide with previous reports. The absence of elevated ACTH levels suggests that other factors contributed to the hypercortisolism in the majority of our patients. We found no relationship between cortisol and blood glucose levels at any time. We cannot therefore state that corticosteroids played a fundamental role in the onset of hyperglycemia in our patients.

#### Growth hormone and insulinlike growth factor-1

GH secretion is induced by stress and the mean serum GH level may be elevated early in the course of trauma or surgery and fall progressively during the first week [25]. Serum IGF-1 levels are almost invariably low in critically ill patients. In acute stress situations, a low IGF-1 level in presence of enhanced GH secretion indicates GH resistance [25]. In our study, GH and IGF-1 levels were initially low and they showed no significant modifications over the course of the study. There was no relationship of the GH and IGF-1 levels with blood glucose, insulin, or C-peptide levels, suggesting that these hormones do not play a role in the onset of hyperglycemia.

#### Thyrotropin and thyroxine

In critically ill patients, T_3_ is typically low on admission, with normal or slightly elevated TSH and T_4_ levels. TSH levels subsequently normalize while T_3_ levels remain low and, in serious diseases such as meningococcal sepsis, T_4_ levels fall [26]. In our study, a large proportion of the children had low TSH levels at the time of diagnosis of hyperglycemia, with normal T_4_ values. However, T_4_ levels fell progressively, probably reflecting the severity of the illness. We are therefore unable to state that there is a clear link between thyroid hormone alterations and the onset of hyperglycemia.

### Relationship of blood glucose levels and treatment with prognosis

Most studies have reported that hyperglycemia occurs early in critical illness and is independently associated with death [1,2,18,19]. However, it is unclear whether this association is indicative of the severity of the underlying illness or that hyperglycemia is itself a risk factor. Other studies in critically ill children have found no association between the blood glucose levels and mortality [27] or length PICU stay [20].

Our study showed that children with persistent severe hyperglycemia had a high mortality (13%)—the overall mortality in our PICU over the study period was 3%. This fact suggest that hyperglycemia is a marker of severity and an indicator of risk of death [28]. However, although the number of patients was low, we did not find a relationship between the absolute blood glucose, C-peptide, or insulin values and mortality. The presence of hyperglycemia would therefore appear to be a marker of severity in the critically ill patient but not itself worsen the prognosis.

On the other hand, we found that the onset of hyperglycemia occurred later in patients that died than in survivors and nonsurvivors presented greater insulin resistance at 72 hours after diagnosis. This could indicate the existence of two types of hyperglycemia in the critically ill patient. An initial, frequently transitory hyperglycemia caused by the stress of admission to the PICU and that is not associated with a poor prognosis, and another of later onset that could reflect more serious metabolic dysregulation with persistent insulin resistance and that is an indicator of mortality.

In our study, the main factors associated with mortality and the length of PICU stay were the clinical severity scores and the presence of severe multiorgan failure with the need for CRRT and ECMO.

Initial studies in critically ill patients found short-term benefits to maintaining normoglycemia [1,29]. However recent studies have found that intensive treatment with insulin does not decrease mortality [6,7,30] and may even be associated with a higher mortality and hypoglycemia [6,7,27,29]. A large proportion of our patients received insulin treatment despite our use of more restrictive indications for insulin treatment and less strict glycemic control objectives. None of our patients developed hypoglycemia, which confirms that insulin treatment with moderate objectives reduces the risk of hypoglycemia.

#### Corticosteroids and prognosis

The higher corticosteroids levels in the patients who died support the hypothesis that these patients had more serious illness and therefore had a more marked adrenal response. Many studies have analyzed the relationship of corticosteroid levels and the adrenal response with the prognosis of septic patients, reporting contradictory results [31,32]. The data from our study only allow us to state that critically ill children with hyperglycemia very often present elevated corticosteroid levels and that patients who die have higher levels than survivors. In critically ill children, both low and elevated corticosteroid levels may be indicators of severity and of risk of death. However, the number of patients who died in our study was small and further studies are necessary to confirm these results.

### Limitations

Our study has certain limitations. The number of patients was low and most of them were in the postoperative period of cardiac surgery. The number of patients could not permit to perform a multiple logistic regression analysis and it is a small sample size to detect a difference in mortality. There was no control group without hyperglycemia to compare hormonal changes. However it is very difficult to achieve a control group with the same characteristics and severity as the study group but without hyperglycemia. To use HOMA in critically ill patients in those glucose and insulin concentrations are not in a steady state and/or are receiving exogenous insulin has limitations because this method has not been verified in these situations and for this reason the interpretation of the results has to be made with caution. The duration of the study was limited, as patients were only studied for 72 hours and we therefore do not know the changes in the hormone levels over the subsequent days, although the majority of the patients achieved adequate glycemic control at 72 hours. PRISM and PIM was measured to evaluate the severity of illness at different times although these scores only have been validated in the first 24 hours of admission. Analytical measures were made at fixed times, independently of the hour of the day and this fact could influence in some hormone values as ACTH or cortisol. Finally, we did not measure other factors that could mediate in the onset of hyperglycemia in the critically ill patient, such as catecholamines, cytokines, glucagonlike peptide-1 or nonesterified fatty acids [33].

## Conclusions

We conclude that critically ill children with severe hyperglycemia initially have reduced β-cell function and insulin sensitivity. β-cell function recovers rapidly but peripheral resistance to insulin persists. There was no relationship between glycemia and the majority of the hormonal changes detected. Patients who died had higher cortisol and growth hormone levels and developed hyperglycemia later than survivors. There was no relationship between glycemia and mortality although the number of patients is too small to detect differences. Length of stay was associated with C-peptide, insulin, and blood glucose levels, insulin resistance, and severity of illness at 24 hours.

## Competing interests

The authors declare that they have no competing interests.

## Authors’ contributions

All authors have made substantial contributions to conception and design, or acquisition of data, or analysis and interpretation of data; have been involved in drafting the manuscript or revising it critically for important intellectual content; and have given final approval of the version to be published. YB and JL-H: conceived the study and participated in the design, data collection and analysis, and drafting of the manuscript. RG, MJS, JC, JU, MB, and AGS participated in the design of the study, data collection and analysis of data, and drafting of the manuscript. NL performed hormonal laboratory analysis and drafted the manuscript. JMB participated in the design of the study and performed the statistical analysis. All authors read and approved the final manuscript.

## Pre-publication history

The pre-publication history for this paper can be accessed here:

http://www.biomedcentral.com/1472-6823/14/25/prepub
